# Assessing Site Specificity of Osteoarthritic Gait Kinematics with Wearable Sensors and Their Association with Patient Reported Outcome Measures (PROMs): Knee versus Hip Osteoarthritis

**DOI:** 10.3390/s21165363

**Published:** 2021-08-10

**Authors:** Corina Nüesch, Petros Ismailidis, David Koch, Geert Pagenstert, Thomas Ilchmann, Anke Eckardt, Karl Stoffel, Christian Egloff, Annegret Mündermann

**Affiliations:** 1 Department of Orthopaedics and Traumatology, University Hospital Basel, 4031 Basel, Switzerland; corina.nueesch@usb.ch (C.N.); petros.ismailidis@usb.ch (P.I.); david.koch@usb.ch (D.K.); karl.stoffel@usb.ch (K.S.); christian.egloff@usb.ch (C.E.); 2 Department of Spine Surgery, University Hospital Basel, 4031 Basel, Switzerland; 3 Department of Biomedical Engineering, University of Basel, 4123 Allschwil, Switzerland; 4 Department of Clinical Research, University of Basel, 4031 Basel, Switzerland; geert.pagenstert@unibas.ch; 5 Department for Sport, Movement and Health, University of Basel, 4052 Basel, Switzerland; 6 Clarahof Clinic of Orthopaedic Surgery, 4058 Basel, Switzerland; 7 ENDO-Team, Hirslanden Klinik Birshof, 4142 Münchenstein, Switzerland; thomas.ilchmann@hirslanden.ch (T.I.); anke.eckardt@hirslanden.ch (A.E.)

**Keywords:** inertial measurement unit, walking, joint motion, gait pattern, function, pain, SPM, HOOS, KOOS

## Abstract

There is a great need for quantitative outcomes reflecting the functional status in patients with knee or hip osteoarthritis (OA) to advance the development and investigation of interventions for OA. The purpose of this study was to determine if gait kinematics specific to the disease—i.e., knee versus hip OA—can be identified using wearable sensors and statistical parametric mapping (SPM) and whether disease-related gait deviations are associated with patient reported outcome measures. 113 participants (N = 29 unilateral knee OA; N = 30 unilateral hip OA; N = 54 age-matched asymptomatic persons) completed gait analysis with wearable sensors and the Knee/Hip Osteoarthritis Outcome Score (KOOS/HOOS). Data were analyzed using SPM. Knee and hip kinematics differed between patients with knee OA and patients with hip OA (up to 14°, *p* < 0.001 for knee and 8°, *p* = 0.003 for hip kinematics), and differences from controls were more pronounced in the affected than unaffected leg of patients. The observed deviations in ankle, knee and hip kinematic trajectories from controls were associated with KOOS/HOOS in both groups. Capturing gait kinematics using wearables has a large potential for application as outcome in clinical trials and for monitoring treatment success in patients with knee or hip OA and in large cohorts representing a major advancement in research on musculoskeletal diseases.

## 1. Introduction

Knee and hip osteoarthritis (OA) are very common degenerative joint diseases that will affect up to 25% of the population during a lifetime [1]. Yet, to date, diagnostics for early OA are lacking and its treatment focuses primarily on alleviating symptoms. The development and investigation of interventions such as pharmaceutics is challenging as clinical trials currently rely on patients reported outcome measures (PROMs) that are subjective, fail to discriminate good from very good condition (low ceiling effect) [2] or are mostly influenced by pain and do not reflect function [3]. Other potential outcomes are objective but have insufficient sensitivity for detecting short term changes (e.g., medical imaging) [4]. Hence, there is a great need for quantitative outcomes that reflect the functional status in patients with knee or hip OA.

The potential of gait analysis in understanding the OA disease process [5]—especially of knee OA [6,7,8,9,10,11]—and its treatment [5,12,13,14,15] has long been recognized. While the literature has largely focused on ambulatory load in the context of knee OA [16], differences in joint motion during walking between patients with (knee or hip) OA and controls have also been observed [5] although reported data are available primarily for the index joint [14,17]. Traditionally, ambulatory loading and joint motion is assessed in dedicated laboratories limiting the application in large scale clinical trials or routine clinical practice. The feasibility of using gait parameters as functional outcome parameters for clinical trials necessitates their simple measurement and specificity to the affected joint.

Technological advancements in recent years produced wearable systems based on inertial measurement units (IMUs) that allow measuring joint kinematics during walking with a major advantage of facilitating assessments within less than 10 min in settings outside of the laboratory such as in outpatient clinics. To date, only few studies have explored their potential for assessing joint kinematics in patients with OA. For instance, for patients with knee OA, differences in stride duration, knee flexion range of motion (ROM) in swing and stance have been assessed using the GaitWalk system [18], kinematic differences at the hip, knee and ankle in the sagittal plane using the H-Gait system [19], and thigh and shank sagittal and coronal, knee sagittal kinematics as well as temporal gait parameters using the GaitSmart^TM^ system [20]. In a series of studies, Ismailidis et al. have shown that known differences in joint kinematics between patients with knee OA [21] or patients with hip OA [22] and healthy controls can be measured using the RehaGait^®^ system, reported kinematic differences not only for the index joint but also to adjacent joints, and confirmed that some of these differences depend on walking speed [23,24].

Lebleu et al. [25] have shown that the limited hip range of motion during walking in patients with knee OA measured using the XSens system can be increased by an intervention, specifically by genicular nerve blockade, supporting the relevance of gait kinematics as a potential quantifiable outcome. Moreover, initial attempts for gait classification for distinguishing patients after hip arthroplasty from healthy controls based on inertial sensor gait kinematics yielded promising results [26]. However, to date there is little evidence on differences in gait patterns in patients with OA and healthy controls depending on whether the knee or the hip is affected [27] and whether this joint specificity can be detected using inertial sensor systems.

Differences in gait patterns can be assessed by considering discrete parameters (e.g., joint angle at initial contact, maximum or minimum joint angles or range of motion) or the entire joint angle trajectories. Statistical parametric mapping (SPM) is a method for examining differences in kinematic trajectories where hypotheses expressed in terms of the model parameters are assessed at each time point with univariate statistics [28]. Because many statistical tests are conducted, adjustments must be made to control for type I errors (false positives) potentially caused by the comparison of joint angles over many time points.

The purpose of this study was to determine if gait kinematics specific to the disease—i.e., knee versus hip OA—can be identified using wearable sensors and SPM and whether disease-related gait deviations are associated with PROMs. We hypothesized that the largest sagittal plane kinematic deviations from asymptomatic controls occur at the index joint with smaller deviations at adjacent joints and joints of the contralateral leg and that these gait deviations from asymptomatic controls are associated with lower clinical scores in patients.

## 2. Methods

### 2.1. Participants

Overall, data of 113 participants were included in the analysis. Of these, 29 had unilateral knee OA and were scheduled to undergo total knee arthroplasty, 30 had unilateral hip OA and were scheduled total hip arthroplasty, and 54 were age-matched asymptomatic persons (Table 1). Subsets of data have been published previously in studies focusing on differences in gait kinematics between limbs in patients with knee OA and compared to healthy controls [21,23] and on differences in gait kinematics between limbs in patients with hip OA and compared to healthy controls [22,24]. For the current analysis, we expanded all groups of participants and focused on detecting differences in joint kinematics between patients with knee OA and patients with hip OA.

Patients were recruited from our hospital and from local orthopedic clinics. Inclusion and exclusion criteria for all groups are listed in Table 2. All subjects gave written informed consent prior to participation. This study was approved by the regional review board and conducted in accordance with the Declaration of Helsinki.

### 2.2. Demographic and Clinical Evaluation

Demographic data including age, sex, body mass, body height, and BMI were recorded for each subject. The subjective severity of the OA was assessed using the PROMs KOOS [29] respective the HOOS [30]. Patients with knee OA completed the KOOS, patients with hip OA the HOOS, and asymptomatic controls both the KOOS and the HOOS. Both tools comprise a total of 40 questions further divided in five subscales consisting of 9/10 questions for pain (KOOS/HOOS Pain), 7/5 for symptoms (KOOS/HOOS Symptoms), 17 for activities of daily living (KOOS/HOOS ADL), 5/4 for sport and recreation (KOOS/HOOS Sport/Rec) and 4 for knee/hip related quality of life (KOOS/HOOS QOL). A total score is calculated for each subcategory with a range from 0 to 100, higher scores representing better conditions. Anteroposterior knee/pelvic X-rays and axial hip X-rays were used to determine the K/L radiographic severity scale of the affected joint of patients [31]. A K/L grade of 1 on the contralateral hip was accepted if it was asymptomatic. The combination of a K/L Grade 3 or 4 and symptoms leading to the decision to perform a TKA/THA was defined as severe knee/hip OA.

### 2.3. Gait Analysis

Gait analysis was performed using the RehaGait^®^ (Hasomed, Magdeburg, Germany) inertial sensor system as described by Nüesch et al. [32]. The system consists of seven sensors (dimensions: 60 mm × 15 mm × 35 mm). Each sensor houses a triaxial accelerometer, gyroscopes and magnetometer (magnetometer data not used in the proprietary software computations). Elastic Velcro straps were used to attach the sensors bilaterally to the lateral foot, lateral lower leg and lateral thigh, and one on the pelvis overlying the 5th lumbar vertebra. For the calibration of the system, participants were asked to stand still for 10 s and then flex the trunk and each hip alternatingly. Sensor data were collected while participants performed a single trial of walking for 20 m at a self-selected speed along a well-lit flat hallway. During this trial, each participant took approximately 15 steps per side.

The spatiotemporal parameters (walking speed, cadence, stride length, stride duration, duration of stance/swing phase and single/double support phase as percentage of the gait cycle) as well as bilateral sagittal plane joint kinematics of the ankle, knee and hip including gait events (e.g., heel strike) were computed using the manufacturer’s proprietary software (Hasomed, Magdeburg, Germany) according to Seel et al. [33]. This process entails the following steps: (1) identification of the joint axis coordinates; (2) matching signs of the joint axis coordinates; (3) identification of the joint position coordinates; and (4) computing joint angle from accelerometer and gyroscope readings [33]. The impact of the heel with the ground during each gait cycle results in a peak of the jerk in the vertical direction [34]. This peak is detected and defines initial contact or heel-strike. At the end of the stance, the motion of the foot relative to the ground is characterized by a distinct change in angular velocity of the foot. This change in angular velocity marks the end of the stance and is used to define toe-off [34].

Bilateral sagittal plane joint kinematics of the ankle, knee and hip including gait events were exported as .csv files. Previous studies have shown good agreement of kinematic parameters with camera-based motion analysis [32], excellent agreement of spatiotemporal parameters with an instrumented treadmill [35,36], and good to excellent reliability in healthy subjects [31,34,35].

### 2.4. Data Processing

Ankle, knee and hip kinematic trajectories were normalized from one heel-strike to the subsequent ipsilateral heel-strike to obtain time normalized kinematic trajectories. The first and last two steps of a trial were excluded resulting in data for, on average, 10 steps per leg and participant.

Kinematic trajectories were time normalized to one gait cycle from heel-strike of one foot to the subsequent heel-strike of the same foot. Dynamic joint range of motion (ROM), minimum and maximum ankle, knee and hip angles during stance and swing were computed using a custom algorithm written in Matlab (MathWorks Inc., Natick, MA, USA) from the time normalized kinematic trajectories for each stride. Average trajectories across all trials for each leg and each participant were computed and used for further analyses.

Mean and standard deviation (SD) kinematic trajectories for ankle, knee and hip angles were computed for each group *g*, joint *j* and side *s* from the time normalized kinematic trajectories of all participants in each group using an in-house algorithm written in Matlab as

(1) $$mean_{j,g,s}\left(t\right)=\frac{\sum_{p=1}^{N}angle_{j,p,s}\left(t\right)}{N}$$

and

(2) $$SD_{j,g,s}\left(t\right)=\sqrt{\frac{\sum_{p=1}^{N}{\left(angle_{j,p,s}\left(t\right)-mean_{j,g,s}\left(t\right)\right)}^{2}}{N}},$$

where *t* is time normalized to one gait cycle, *p* is the participant number, *N* the number of participants in group *g* and side *s* affected or unaffected in patients and left or right in controls, respectively (affected side of patients was matched with left side of controls, and unaffected side of patients was matched with right side of controls).

Deviations from the mean of the control group were computed for the affected side of each patient *p*, joint *j* and time point *t* as

(3) $$\Delta angle_{j,p}\left(t\right)=angle_{j,p}\left(t\right)-mean_{j,controls,\;left}\left(t\right)$$

### 2.5. Statistical Analyses

The required number of subjects was calculated based on group differences in discrete parameters reported in the literature [16,37] and based on previous comparisons between patients with knee OA [21] or patients with hip OA [22] versus asymptomatic controls. Sample size estimation revealed that 14 participants were required to detect an expected difference in knee ROM with an effect size of at least 1.12 with 80% power at a 5% significance level. Because it was expected that SPM is at least as sensitive in detecting differences between groups as 0-dimensional methods, we deemed the available dataset as sufficient for the planned analyses.

Because all demographic parameters were normally distributed, analysis of variance (ANOVA) with independent sample *t*-tests as posthoc tests were performed to detect statistically significant group differences in demographic parameters, and parametric descriptive statistics included mean and standard deviation for each group. The significance level was set a priori to 0.05 for the ANOVA and to 0.05/3 for posthoc tests with Bonferroni adjustment. Mean and standard deviations for discrete ankle, knee and hip kinematic parameters were computed and are presented in Table S1 to facilitate comparison to the literature.

For the kinematic trajectories, we tested the null hypothesis that there are no differences in 1D kinematic data between patients with knee OA, patients with hip OA and asymptomatic control subjects. We examined the entire time normalized mean kinematic trajectories using SPM. All SPM analyses were conducted in Matlab (MathWorks Inc., Natick, MA, USA) using the opensource software package spm1D 0.4 (www.spm1d.org; accessed on 15 January 2021) [28]. Between-group statistical analyses were conducted as described by Pataky [28]. Briefly, an ANOVA was performed for each joint to detect an overall group effect. Independent sample *t*-tests were performed to compare pairs of groups in a posthoc analysis. The null hypothesis was rejected if the experimentally computed *t*-value for trajectory 1D data exceeded the critical value that was calculated based on Gaussian random field theory. The significance level was set to 0.05 for the ANOVA of the SPM analysis and to 0.05/3 for the posthoc *t*-tests for independent samples with Bonferroni adjustment.

Statistically significant associations between KOOS and HOOS subscores and kinematic trajectories among patients with knee OA and controls and among patients with hip OA and controls, respectively, were evaluated through scalar trajectory linear regression tests [38] (documented in www.spm1D.org, v0.4, accessed on 1 August 2021). The analyses were conducted for each joint separately. For each SPM analysis, the t statistic was computed at each point in the time series. We visualized the results as mean ±1 standard deviation of the joint angles (vertical axis) time-normalized to one gait cycle), SPM statistic (vertical axis) time-normalized to one gait cycle and mean difference in joint angles between groups (vertical axis) time-normalized to one gait cycle. To allow for the visualization of group differences in the context of variability in joint angles in the control group, we also included ±1 standard deviation of the joint angles of the control group across the gait cycle in the mean difference in joint angle graphs. The significance level was set a priori to 0.05.

## 3. Results

Descriptive statistics for spatiotemporal parameters and 95% confidence intervals for differences between groups are given in Table S1.

### 3.1. Overall Group Effect in Joint Kinematics

**Figure 1:**
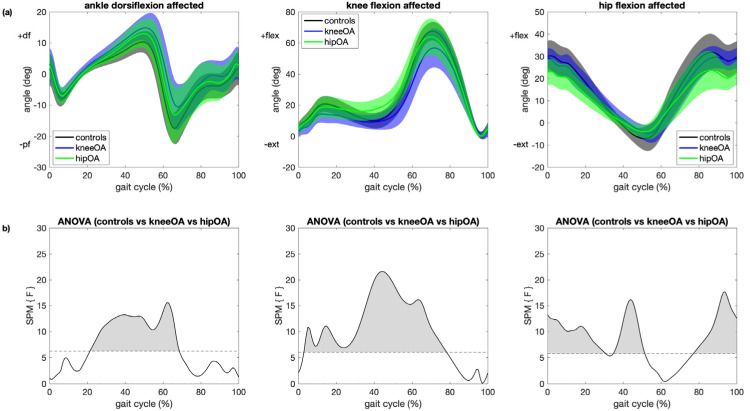
Comparison between all participant groups. (**a**) Mean and 1 standard deviation joint angle trajectories for ankle dorsiflexion (left), knee flexion (center) and hip flexion (right) angle for the affected/left leg for patients with knee OA (blue), patients with hip OA (green) and controls (black); (**b**) results of statistical parametric mapping with analysis of variance (SPM{F}) where regions with statistically significant difference between all groups are indicated as grey regions (*p* < 0.05).

SPM with ANOVA revealed statistically significant differences in ankle, knee and hip kinematic trajectories between the affected/left side of all groups for most of the gait cycle (Figure 1). Ankle dorsiflexion differed between groups during midstance and terminal stance with no significant differences during early stance and swing. Knee flexion differed between groups for the entire stance phase and the first half of swing. Hip flexion differed between groups for the entire gait cycle except during the first half of swing. Descriptive statistics of selected discrete ankle, knee and hip kinematic parameters and 95% confidence intervals for differences between groups are given in Table S1.

Fewer differences in knee and hip kinematic trajectories were observed between the unaffected/right side of all groups (Figure S1). Knee flexion differed between groups only during the first half of swing. Hip flexion differed between groups only during terminal stance and pre-swing.

### 3.2. Patients with OA Versus Controls

**Figure 2:**
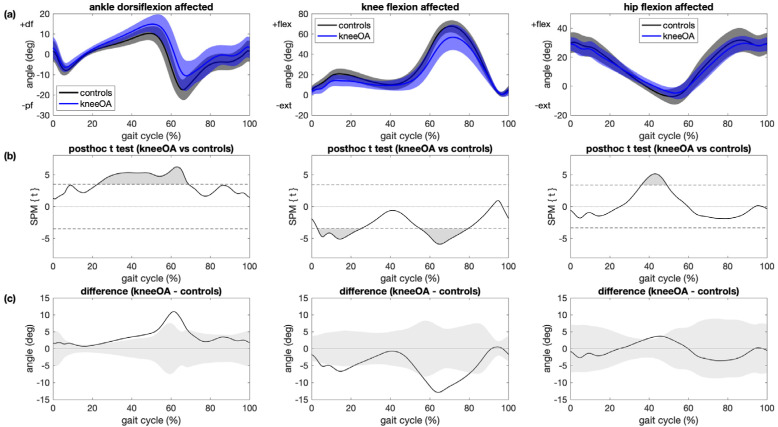
Comparison between patients with knee OA and controls. (**a**) Mean and 1 standard deviation joint angle trajectories for ankle dorsiflexion (left), knee flexion (center) and hip flexion (right) angle for the affected/left leg for patients with knee OA (blue) and controls (black); (**b**) results of statistical parametric mapping with posthoc *t* tests (SPM{t}) where regions with statistically significant difference between groups are indicated as grey regions (*p* < 0.05/3); (**c**) mean joint angle deviation from the control group for patients with knee OA where grey areas illustrate ±1 SD of the joint angles of the control group.

Ankle, knee and hip kinematic trajectories for patients with knee OA and controls are shown in Figure 2. Main kinematic differences between the affected leg in patients with knee OA and controls were greater ankle dorsiflexion during midstance, terminal stance and pre-swing (+12°, *p* < 0.001), less knee flexion during weight acceptance (−7°, *p* < 0.001) and in the first half of swing (−13°, *p* < 0.001) and less hip extension during terminal stance (−4°, *p* = 0.002). Kinematic differences between the contralateral leg in patients with knee OA and controls included greater ankle dorsiflexion during midstance through early swing (+13°, *p* < 0.001), less knee flexion during early swing (−13°, *p* < 0.001) and less hip extension during terminal stance (−5°, *p* < 0.001; Figure S2). The reported differences in brackets are maximum angles in the areas that have significant differences between different groups.

**Figure 3:**
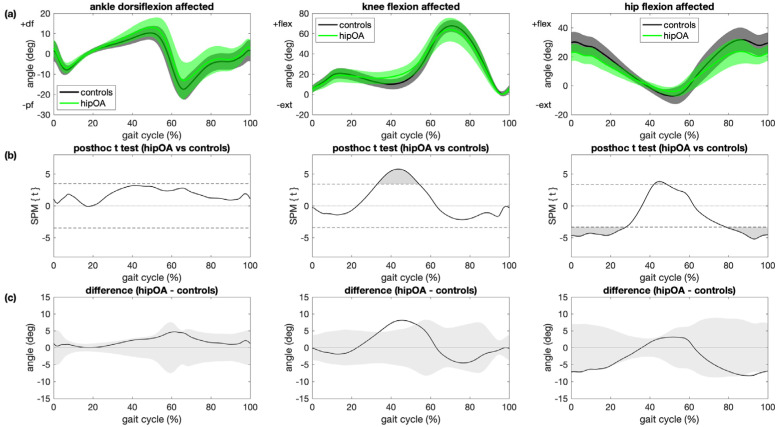
Comparison between patients with hip OA and controls. (**a**) Mean and 1 standard deviation joint angle trajectories for ankle dorsiflexion (left), knee flexion (center) and hip flexion (right) angle for the affected/left leg for patients with hip OA (green) and controls (black); (**b**) results of statistical parametric mapping with posthoc *t* tests (SPM{t}) where regions with statistically significant difference between groups are indicated as grey regions (*p* < 0.05/3); (**c**) mean joint angle deviation from the control group for patients with hip OA where grey areas illustrate ±1 SD of the joint angles of the control group.

In patients with hip OA, differences in ankle dorsiflexion from controls were not statistically significant (Figure 3). Main kinematic differences between the affected leg in patients with hip OA and controls were greater knee flexion during terminal stance (+8°, *p* < 0.001), less hip flexion during weight acceptance and late swing (−8°, *p* < 0.001) and less hip extension during mid- to terminal stance (−3°, *p* = 0.010; Figure 3). Kinematic differences between the contralateral leg in patients with hip OA and controls included greater ankle dorsiflexion during pre- and early swing (+9°, *p* < 0.001), no differences at the knee and less hip extension during terminal stance (−3°, *p* = 0.002; Figure S3). The reported differences in brackets are maximum angles in the areas that have significant differences between different groups.

### 3.3. Patients with Hip OA Versus Patients with Knee OA

**Figure 4:**
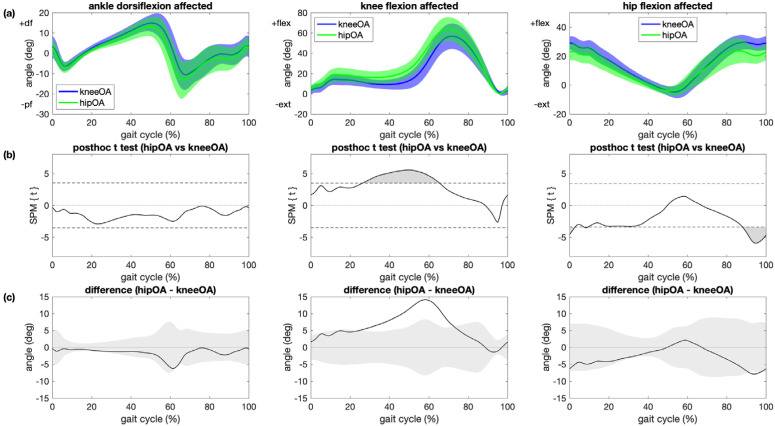
Comparison between patients with knee OA and patients with hip. (**a**) Mean and 1 standard deviation joint angle trajectories for ankle dorsiflexion (left), knee flexion (center) and hip flexion (right) angle for the affected leg for patients with knee OA (blue) and patients with hip OA (green); (**b**) results of statistical parametric mapping with posthoc *t* tests (SPM{t}) where regions with statistically significant difference between groups are indicated as grey regions (*p* < 0.05/3); (**c**) mean joint angle deviation from patients with knee OA for patients with hip OA where grey areas illustrate ±1 SD of the joint angles of the control group.

Differences in ankle dorsiflexion between patients with hip OA and patients with knee OA were not statistically significant (Figure 4). Main kinematic differences between the affected leg in patients with hip OA and patients with knee OA were greater knee flexion during midstance, terminal stance and pre-swing (+14°, *p* < 0.001) and less hip extension during late swing (−8°, *p* = 0.003) and initial contact (−6°, *p* = 0.015; Figure 4). Kinematic trajectories for the contralateral ankles, knees and hips did not differ significantly between patients with hip OA and patients with knee OA (Figure S4). The reported differences in brackets are maximum angles in the areas that have significant differences between different groups.

### 3.4. Association between Gait Deviations from Controls and PROMs

#### 3.4.1. Knee OA

**Figure 5:**
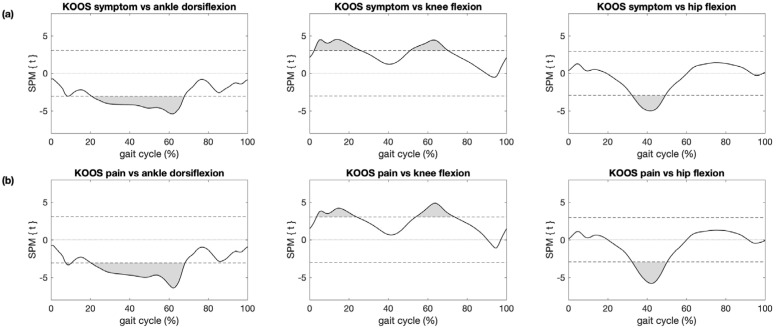
Association between KOOS subscores and kinematic trajectories. Results of statistical parametric mapping with regression analysis (SPM{t}) are shown for (**a**) the KOOS subscore symptoms and (**b**) the KOOS subscore pain and the ankle (left), knee (center) and hip (right) of the affected/left leg where grey areas illustrate regions with statistically significant relationships between KOOS subscores and kinematics (*p* < 0.05).

The association between KOOS subscores and kinematic trajectories among patients with knee OA and controls were significant for ankle dorsiflexion during midstance, terminal stance and pre-swing knee flexion during weight acceptance and in the first half of swing and hip flexion during terminal stance (Figure 5 and Figure S5). These regions coincided with regions where significant differences in kinematic trajectories between patients with knee OA and controls were observed (Figure 2).

#### 3.4.2. Hip OA

**Figure 6:**
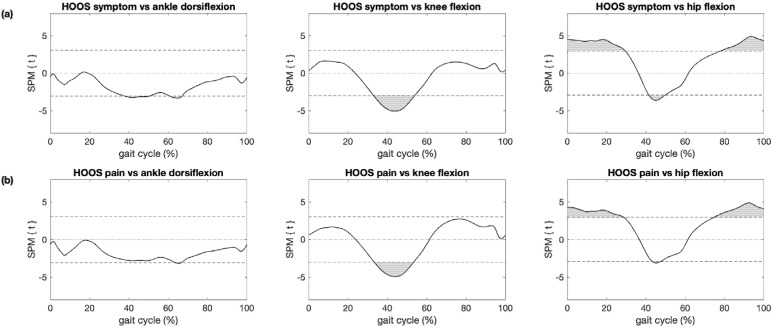
Association between HOOS subscores and kinematic trajectories. Results of statistical parametric mapping with regression analysis (SPM{t}) are shown for (**a**) the HOOS subscore symptoms and (**b**) the HOOS subscore pain and the ankle (left), knee (center) and hip (right) of the affected/left leg where grey areas illustrate regions with statistically significant relationships between HOOS subscores and kinematics (*p* < 0.05).

The association between HOOS subscores and kinematic trajectories among patients with hip OA and controls were significant for ankle dorsiflexion during terminal stance (HOOS symptoms and HOOS QOL only) and early swing (HOOS symptoms, HOOS pain, HOOS ADL and HOOS QOL), knee flexion during terminal stance and hip flexion during weight acceptance, midstance (except HOOS sport/rec) and late swing (Figure 6 and Figure S6). Except for the ankle, these regions coincided mostly with regions where significant differences in kinematic trajectories between patients with hip OA and controls were observed (Figure 3).

## 4. Discussion

The purpose of this study was to determine if gait kinematics specific to knee versus hip OA can be identified using wearable sensors and SPM and whether disease-related gait deviations are associated with PROMs. We identified clear differences in knee and hip kinematics between patients with knee OA and patients with hip OA. Moreover, patients with knee OA showed deviations from controls in ankle, knee and hip kinematic patterns in their affected leg with fewer deviations in the contralateral leg. Patients with hip OA showed deviations from control in knee and hip kinematics of the affected with fewer deviations in the contralateral leg. These results confirm our hypothesis that the largest kinematic deviations from asymptomatic controls occur at the index joint with smaller deviations at adjacent joints and joints of the contralateral leg. The observed deviations in ankle, knee and hip kinematics from controls were associated with PROMs in both groups.

To date, a direct comparison of gait kinematics between patients with knee OA and patients with hip OA is scarce and limited to discrete gait parameters [27]. A wealth of evidence on differences in joint kinematics during walking in the index joint of patients with knee or hip OA compared to healthy controls has been published. According to a systematic review [16], these differences range between 2.5° and 10.9° and in some studies up to 23° in patients with knee OA. Main differences include lower knee ROM and peak flexion during swing and stance, especially in patients with severe knee OA [27,39,40] and smaller knee flexion-extension ROM over the entire gait cycle [39]. More recently, van der Straaten et al. [17] reported in a systematic review of the literature on mobile kinematic assessments that in patients with knee OA, the knee flexion ROM during stance and swing is about 10° smaller than that in healthy controls. Surprisingly, few studies have investigated kinematic differences at the ankle or hip between patients with knee OA and controls [16,27,41,42]. While some studies found no difference in ankle or hip kinematics between patients with knee OA, others reported smaller ankle ROM, greater ankle dorsiflexion or lower peak hip extension in patients with severe knee OA [21,27,39,42].

The main reported differences in discrete parameters describing hip kinematics in patients with hip OA compared to controls include smaller hip flexion ROM with lower [6,36,40,43] or no differences in peak flexion [27] and lower peak extension [6,36,40,43]. Considering the entire kinematic trajectories, Ardestani et al. [40] and Porta et al. [41] observed smaller hip flexion in early stance and hip extension between 30 and 70% of the gait cycle. Only a few studies on gait in hip OA reported data for the ankle or knee. Of these, some reported no significant differences in knee ROM [6], while others described a reduction in knee flexion ROM [22,27,36,40,44,45] and greater [6,22,45] or comparable [27] peak ankle dorsiflexion in the affected leg in patients with hip OA compared to controls.

The results of our study regarding deviation in ankle, knee and hip kinematics in patients with knee OA and in patients with hip OA compared to controls confirm the literature. Based on the data reported in the literature and presented in this paper for each group, one would expect similarities between groups in greater peak ankle dorsiflexion and reduced knee flexion ROM during stance and hip extension during stance. Expected discrepancies would be greater ankle dorsiflexion ROM, peak knee flexion during stance and swing, knee flexion ROM during swing and reduced peak hip flexion in patients with hip OA compared to patients with knee OA. Indeed, the comparison of ankle, knee and hip kinematics between patients with hip OA and patients with knee OA in our study revealed no differences in ankle plantar/dorsiflexion between groups and greater knee flexion during midstance, terminal stance and pre-swing and less hip extension during late swing and initial contact in patients with hip OA than in patients with knee OA. These results represent strong evidence for the presence of disease-related gait deviations from controls that are specific to the affected joint and confirm results of a previous study [27]. Based on our results it seems feasible that data obtained using wearable gait analysis systems may contribute to a better understanding of the primary affected site and may be sensitive to changes in function specific to the affected joint. Based on our clinical experience, many patients affected by OA also have other ailments such as low back problems (e.g., spinal stenosis) or are affected at multiple joints. Hence, the potential of wearable data should be further explored in studies involving patients with degenerative joint disease of varying severity and different sites.

Deviations in ankle, knee and hip kinematics from controls were associated with the subscores of the KOOS and HOOS in patients with knee OA and patients with hip OA, respectively. Interestingly, the associations were significant in those regions of the kinematic trajectories, where trajectories differed significantly from patterns of the control group. These results suggest that patients with knee or hip OA with greater subjective pain, symptoms or functional limitation also have greater deviations in gait patterns from age-matched controls. Our data support previous reports of the association of mechanical factors and pain in patients with knee OA using discrete parameters [42] but contradict results reported by Astephen Wilson et al. [43], who reported no association between knee flexion angle trajectories (and knee adduction moment) and pain assessed using principal component analysis. In a more recent study on a very small group of patients, Bensalma et al. [44] explored the value of different discrete knee kinematic parameters in explaining differences in pain in patients with knee OA using canonical correlation analysis and identified groups of parameters that may be of clinical relevance. However, these studies only considered ambulatory knee biomechanics and did not consider gait deviations at other joints. Rosenlund et al. [45] computed the gait deviation index from nine continuous variables including ankle, knee and hip kinematics and found that patients with hip OA with greater gait deviation from controls had worse HOOS subscore values. While the strength of that study is that the kinematics of the entire limb was considered, the computation obscures the contribution of each of the joints to the gait deviation. Moreover, Tateuchi et al. [46] showed that limited hip extension during gait is one of the factors that was related to a deterioration in physical function in patients with hip OA, suggesting that deviations from kinematics in healthy persons may not only reflect the functional status of patients but are also valuable for predicting outcome.

All of these studies have in common that gait kinematics were collected using laboratory-based motion analysis systems. In our study, we provided first evidence for an association of ankle, knee and hip kinematics obtained using wearable sensors and PROMs. In this study, we chose to explore our data using SPM. While we acknowledge the current large push—in academia and industry—towards big data approaches for evaluating motion data using wearables and artificial intelligence, with the current study we would like to emphasize the importance of considering parameters with a functional, biomechanical and clinical meaning that capture joint function and biomechanics in the context of disease development, progression and treatment. The data presented here suggest that joint kinematics may be a suitable objective and measurable data type that should be explored further in studies on early OA, diagnostics, clinical exams and as outcomes for clinical trials on various conservative, surgical or pharmaceutical therapies. Moreover, our data clearly show the effect of OA in one joint on its adjacent joints, and hence focusing on the index joint may obscure important functional changes in overall ambulatory biomechanics.

A major strength of this study is the consideration of kinematic trajectories rather than only discrete parameters allowing to identify the phases of gait with the most relevant kinematic deviations. This knowledge may be helpful for the therapist or clinician to focus specifically on these phases during clinical exams without instrumentation. Moreover, in contrast to most previous studies, we included data on joints adjacent to the index joint. One of the limitations of this cross-sectional study is that we included only patients with severe OA awaiting joint replacement surgery and did not investigate the sensitivity of deviations in ankle, knee and hip kinematics to intervention, which should be explored in future studies. Although patients with OA had a significantly higher BMI, the mean differences in BMI between patients and controls were 2.6 kg/m^2^, and hence we decided not to account for this discrepancy in our analyses. This factor should be considered in future trials in large cohorts. Further, while we relied on the manufacturer’s algorithm to compute kinematic trajectories, previous studies using the same system have produced consistent and reliable results hence supporting the relevance of the data presented here. Finally, we limited our analyses to sagittal plane kinematics. Further gait kinematic deviations may be present in the frontal and transverse planes. These and their association with PROMs should be further explored in future studies.

## 5. Conclusions

Known kinematic patterns associated with knee or hip OA can not only be measured with a wearable inertial sensor system, but these gait alterations are also specific to knee or hip OA, respectively. Main kinematic differences to asymptomatic control subjects were observed at the respective index joint with lesser compensation at adjacent joints. These results point towards the potential of using gait analysis with wearable inertial sensors to help in the diagnosis of the primary disease site. This study represents an important step towards future use of kinematic gait parameters as outcome in clinical trials and for monitoring treatment success in patients with knee or hip OA. Moreover, observed changes at adjacent joints emphasize the importance of assessing overall kinematic changes to recognize the potential of developing OA at adjacent or contralateral joints because of compensatory mechanisms. Clearly, capturing gait kinematics using wearables as those utilized in this study have a great potential for application in large cohorts, representing a major advancement in movement science and research on musculoskeletal diseases. Assessing these data can now be easily implemented in routine clinical practice complementing and objectifying PROMs.

## Figures and Tables

**Table 1:** Demographics of patients with knee OA, patients with hip OA and asymptomatic controls. Values for personal parameters are shown as mean (1 standard deviation).

Parameter	Patients with Knee OA	Patients with Hip OA	Asymptomatic Controls
N	29	30	54
Sex (female/male)	16/13	18/12	23/31
Age (years)	67.2 (8.5)	64.9 (11.6)	66.4 (7.9)
Body height (m)	1.70 (0.10)	1.72 (0.08)	1.70 (0.09)
Body mass (kg)	80.6 (14.4) ^1^	80.6 (12.8) ^1^	73.1 (13.5)
Body mass index (kg/m^2^)	27.9 (3.9) ^1^	27.1 (3.1) ^1^	25.3 (4.0)
KOOS			
Symptoms	50.1 (19.9) ^1^		95.4 (6.1)
Pain	47.5 (17.7) ^1^		97.6 (3.9)
ADL	52.5 (21.9) ^1^		98.2 (3.0)
Sport/rec	28.2 (19.8) ^1^		94.9 (9.1)
QOL	25.6 (19.0) ^1^		95.5 (8.8)
HOOS			
Symptoms		48.3 (16.8) ^1^	96.9 (5.6)
Pain		50.9 (14.9) ^1^	98.2 (4.4)
ADL		53.9 (17.3) ^1^	98.6 (3.5)
Sport/rec		36.1 (20.0) ^1^	98.0 (5.0)
QOL		27.8 (16.4) ^1^	97.3 (7.2)

^1^ significantly different from controls (*t*-tests, *p* < 0.05/3). OA—osteoarthritis; KOOS—knee osteoarthritis outcome score; HOOS—hip osteoarthritis outcome score; a normalized score (100 indicating no symptoms and 0 indicating extreme symptoms) is calculated for each subscale; ADL—function in activities of daily living; sport/rec—function in sport and recreation; QOL—knee/hip related quality of life.

**Table 2:** Inclusion and exclusion criteria.

Criteria	Patients with Knee OA	Patients with Hip OA	Asymptomatic Controls
Inclusion criteria	Age ≥ 30 years Diagnosed unilateral OA of the knee Radiographic severity Kellgren/Lawrence (K/L) 3 or 4 Scheduled for primary total knee arthroplasty	Age ≥ 30 years Diagnosed unilateral OA of the hip Radiographic severity Kellgren/Lawrence (K/L) 3 or 4 Scheduled for primary total hip arthroplasty	Age ≥ 30 years No clinical diagnosis of OA, rheumatoid arthritis or history of knee or hip trauma or pain at the time of the measurement
Exclusion criteria	BMI > 35 kg/m^2^ Use of walking aids; neuromuscular disorders affecting gait Current pain of the lower back Inability to follow procedures due to psychological disorders or dementia	BMI > 35 kg/m^2^ Use of walking aids; neuromuscular disorders affecting gait Current pain of the lower back Inability to follow procedures due to psychological disorders or dementia	BMI > 35 kg/m^2^ Use of walking aids; neuromuscular disorders affecting gait Current pain of the lower back Inability to follow procedures due to psychological disorders or dementia KOOS or HOOS <90 in the pain subcategory

OA—osteoarthritis; BMI—body mass index; KOOS—knee osteoarthritis outcome score; HOOS—hip osteoarthritis outcome score.

## Data Availability

All data underlying the analyses are presented within this document. Raw data can be obtained from the authors upon request.

## Supplementary Materials

The following are available online at https://www.mdpi.com/article/10.3390/s21165363/s1, Table S1: Spatiotemporal and discrete ankle, knee and hip kinematic parameters, Figure S1: Comparison between all participant groups for the contralateral/right leg, Figure S2: Comparison between patients with knee OA and controls for the contralateral/right leg, Figure S3: Comparison between patients with hip OA and controls for the contralateral/right leg, Figure S4: Comparison between patients with knee OA and patients with hip OA for the contralateral/right leg, Figure S5: Association between KOOS subscores and kinematic trajectories., Figure S6: Association between HOOS subscores and kinematic trajectories.
