# Supplementary material for: Assessing Site Specificity of Osteoarthritic Gait Kinematics with Wearable Sensors and Their Association with Patient Reported Outcome Measures (PROMs): Knee versus Hip Osteoarthritis
Source: Sensors (Basel). 2021 Aug 10;21(16):5363. doi: 10.3390/s21165363 (PMC8398113; doi:10.3390/s21165363)
Supplement: Supplementary file 1 [file sensors-21-05363-s001.zip › Figure S2.pdf]

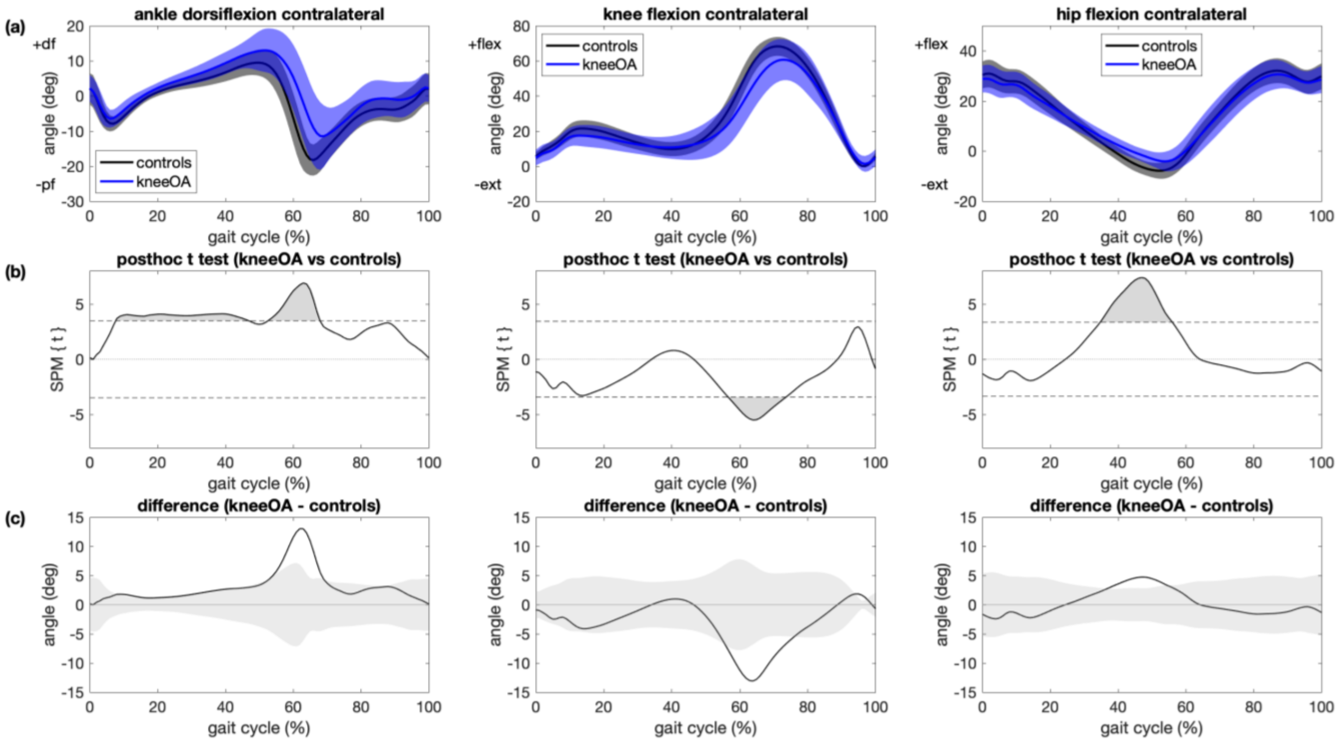

Figure S2. Comparison between patients with knee OA and controls for the contralateral/right leg. (a) Mean and 1 standard deviation joint angle trajectories for ankle dorsiflexion (left), knee flexion (center) and hip flexion (right) angle for the unaffected/right leg for patients with knee OA (blue) and controls (black); **(b)** results of statistical parametric mapping with posthoc t tests (SPM{t}) where regions with statistically significant difference between groups are indicated as grey regions ($P < 0.017$); **(c)** mean joint angle deviation from the control group for patients with knee OA where grey areas illustrate ± 1 SD of the joint angles of the control group.
