# Supplementary material for: Assessing Site Specificity of Osteoarthritic Gait Kinematics with Wearable Sensors and Their Association with Patient Reported Outcome Measures (PROMs): Knee versus Hip Osteoarthritis
Source: Sensors (Basel). 2021 Aug 10;21(16):5363. doi: 10.3390/s21165363 (PMC8398113; doi:10.3390/s21165363)
Supplement: Supplementary file 1 [file sensors-21-05363-s001.zip › Figure S4.pdf]

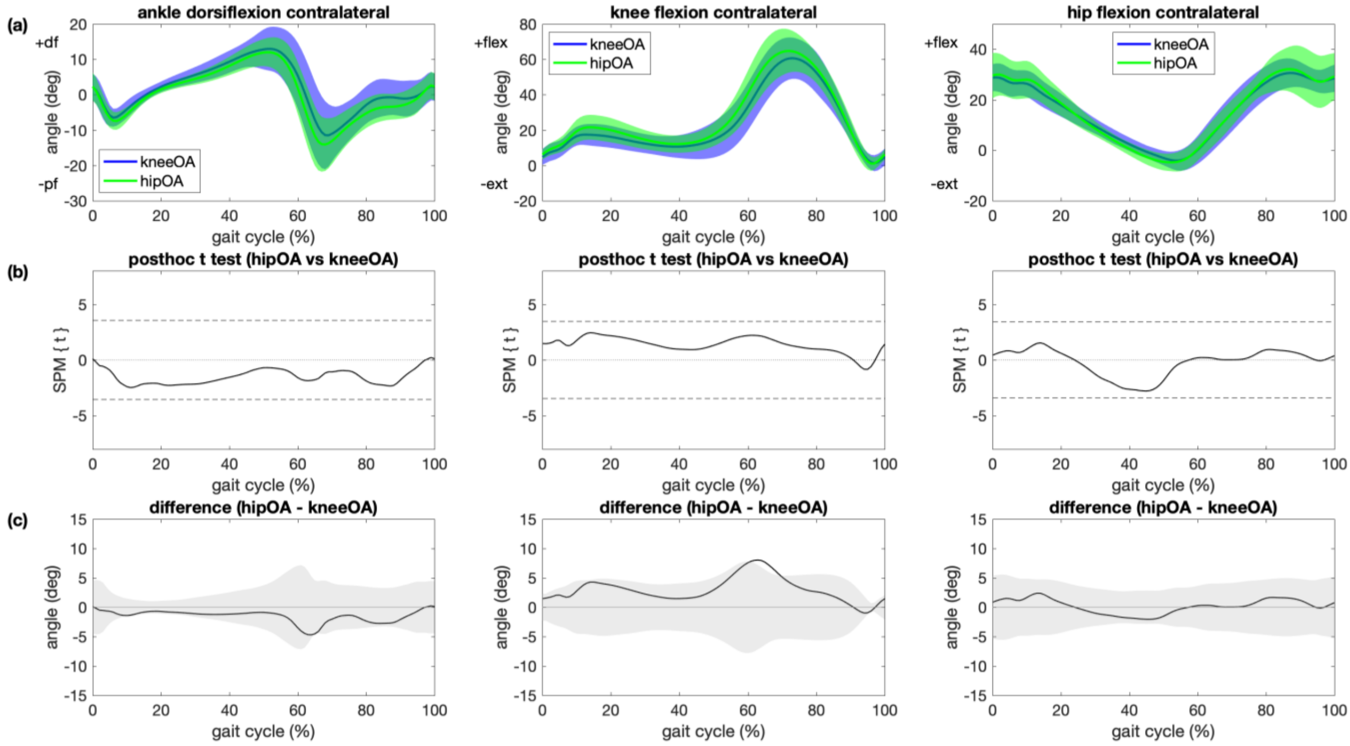

Figure S4. Comparison between patients with knee OA and patients with hip for the contralateral/right leg. (**a**) Mean and 1 standard deviation joint angle trajectories for ankle dorsiflexion (left), knee flexion (center) and hip flexion (right) angle for the affected leg for patients with knee OA (blue) and patients with hip OA (green); (**b**) results of statistical parametric mapping with posthoc t tests (SPM{t}) where regions with statistically significant difference between groups are indicated as grey regions ($P < 0.017$); (**c**) mean joint angle deviation from patients with knee OA for patients with hip OA where grey areas illustrate ± 1 SD of the joint angles of the control group.
