# Supplementary material for: Assessing Site Specificity of Osteoarthritic Gait Kinematics with Wearable Sensors and Their Association with Patient Reported Outcome Measures (PROMs): Knee versus Hip Osteoarthritis
Source: Sensors (Basel). 2021 Aug 10;21(16):5363. doi: 10.3390/s21165363 (PMC8398113; doi:10.3390/s21165363)
Supplement: Supplementary file 1 [file sensors-21-05363-s001.zip › Figure S6.pdf]

sensors MDPI

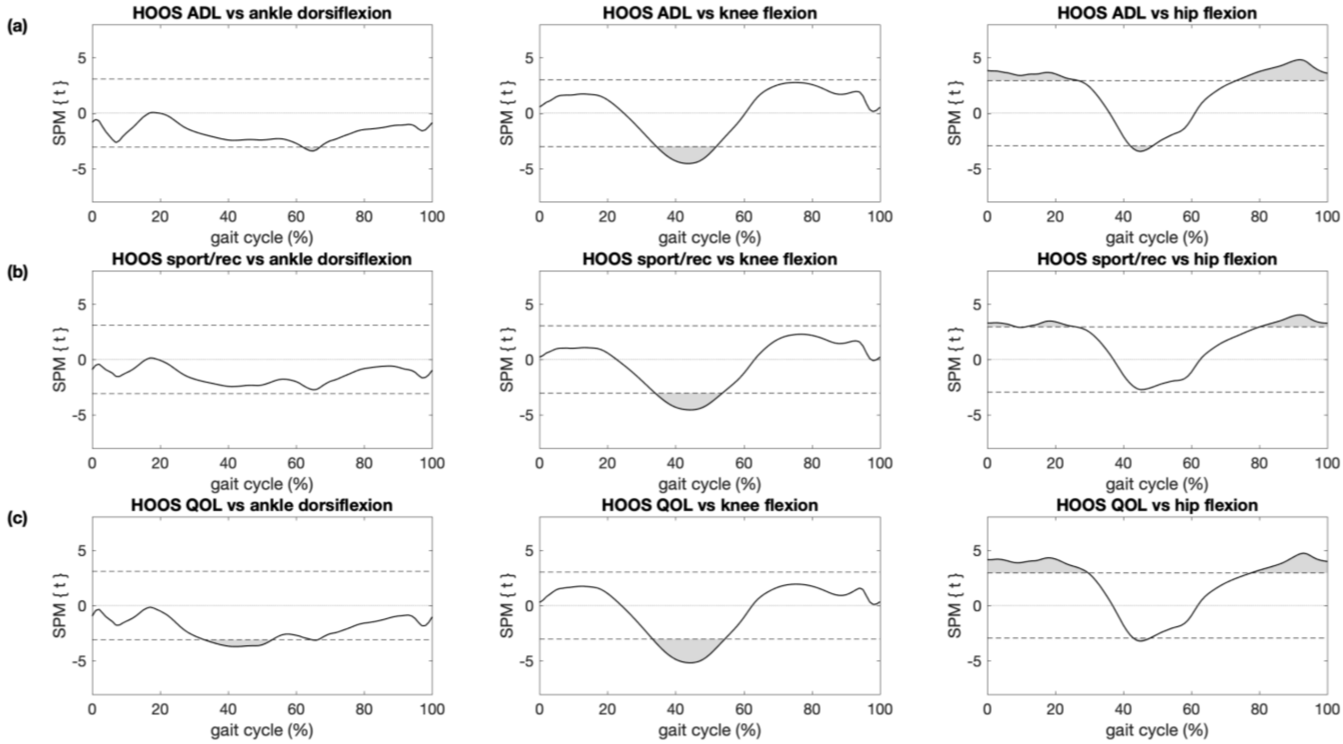

Figure S5. Association between HOOS subscores and kinematic trajectories. Results of statistical parametric mapping with regression analysis (SPM{t}) are shown for (**a**) the HOOS subscore activity of daily living (ADL), **(b)** the HOOS subscore sport and recreation (sport/rec) and **(c)** the HOOS subscore quality of life (QOL) and the ankle (left), knee (center) and hip (right) of the affected/left leg where grey areas illustrate regions with statistically significant relationships between HOOS subscores and kinematics (P<0.05).
