# Supplementary material for: Assessing Site Specificity of Osteoarthritic Gait Kinematics with Wearable Sensors and Their Association with Patient Reported Outcome Measures (PROMs): Knee versus Hip Osteoarthritis
Source: Sensors (Basel). 2021 Aug 10;21(16):5363. doi: 10.3390/s21165363 (PMC8398113; doi:10.3390/s21165363)
Supplement: Supplementary file 1 [file sensors-21-05363-s001.zip › Table S1.pdf]

sensors

MDPI

Table S1. Spatiotemporal and discrete ankle, knee and hip kinematic parameters in patients with knee osteoarthritis (OA), patients with hip OA and asymptomatic controls. Data are shown as mean (1 standard deviation). 95% confidence intervals (CI) of the difference between groups are shown.

Parameter	Patients with knee OA N=29	Patients with hip OA N=30	Asymptomatic controls N=54	Knee OA - controls 95% CI	Hip OA - controls 95% CI	Hip OA - knee OA 95% CI
Spatiotemporal parameters						
Walking speed (m/s)	0.98 (0.21)	1.08 (0.22)	1.25 (0.15)	[-0.35; -0.19]	[-0.26; -0.10]	[-0.21; 0.02]
Stride time (s)	1.184 (0.144)	1.150 (0.142)	1.107 (0.137)	[0.013; 0.141]	[-0.020; 0.105]	[-0.040; 0.109]
Stride length (m)	1.14 (0.20)	1.18 (0.17)	1.34 (0.13)	[-0.27; -0.12]	[-0.22; -0.09]	[-0.13; 0.06]
Cadence (steps/minute)	102.7 (10.1)	108.6 (11.6)	112.6 (8.5)	[-14.0; -5.7]	[-8.4; 0.4]	[-11.6; -0.2]
Stance phase affected/left side (% gait cycle)	65.0 (3.1)	62.9 (2.7)	62.4 (1.9)	[1.50; 3.70]	[-0.50; 1.52]	[0.59; 3.59]
Stance phase unaffected/right side (% gait cycle)	64.7 (4.7)	65.0 (4.7)	62.5 (1.8)	[0.71; 3.59]	[1.06; 3.94]	[-2.81; 2.11]
Swing phase affected/left side (% gait cycle)	35.0 (3.1)	37.1 (2.67	37.6 (1.9)	[-3.70; -1.50]	[-1.52; 0.50]	[-3.59; -0.59]
Swing phase unaffected/right side (% gait cycle)	35.4 (4.7)	35.0 (4.7)	37.5 (1.8)	[-3.59; -0.71]	[-3.94; -1.06]	[-2.11; 2.81]
Single support phase affected/left side (% gait cycle)	35.6 (4.4)	35.1 (4.4)	37.6 (1.7)	[-3.31; -0.61]	[-3.76; -1.06]	[-1.84; 2.75]
Single support phase unaffected/right side (% gait cycle)	35.4 (2.8)	37.9 (4.0)	37.6 (1.9)	[-3.25; -1.18]	[-1.04; 1.53]	[-4.25; -0.67]
Double support phase affected/left side (% gait cycle)	15.5 (5.1)	16.4 (11.1)	12.6 (1.7)	[1.45; 4.49]	[0.70; 6.91]	[-5.35; 3.68]
Double support phase unaffected/right side (% gait cycle)	17.4 (11.9)	13.5 (4.8)	12.8 (2.3)	[1.25; 8.02]	[-0.79; 2.35]	[-0.85; 8.57]
Double support time affected/left side (s)	0.184 (0.070)	0.190 (0.134)	0.153 (0.098)	[-0.009; 0.073]	[-0.014; 0.088]	[-0.062; 0.050]
Double support time unaffected/right side (s)	0.202 (0.126)	0.157 (0.067)	0.144 (0.037)	[0.020; 0.094]	[-0.010; 0.036]	[-0.008; 0.097]

Parameter	Patients with knee OA N=29	Patients with hip OA N=30	Asymptomatic controls N=54	Knee OA - controls 95% CI	Hip OA - controls 95% CI	Hip OA - knee OA 95% CI
Ankle kinematics						
Dorsiflexion at initial contact affected/left side (°)	3.2 (5.3)	2.8 (4.3)	1.6 (5.1)	[-0.9; 4.0]	[-1.0; 3.4]	[-2.2; 3.0]
Dorsiflexion at initial contact unaffected/right side (°)	2.2 (3.8)	2.3 (3.9)	2.1 (4.5)	[-1.8; 2.1]	[-1.7; 2.2]	[-2.1; 1.9]
Maximum plantarflexion during weight acceptance affected/left side (°)	8.0 (2.3)	8.0 (2.6)	9.0 (3.2)	[-0.4; 2.4]	[-0.4; 2.3]	[-1.2; 1.4]
Maximum plantarflexion during weight acceptance unaffected/right side (°)	7.9 (2.3)	8.3 (2.4)	8.9 (2.8)	[-0.3; 2.1]	[-0.6; 1.8]	[-0.9; 1.6]
Maximum dorsiflexion during terminal stance affected/left side (°)	15.8 (3.9)	14.1 (4.8)	10.8 (3.4)	[3.3; 6.7]	[1.5; 5.1]	[-0.6; 4.1]
Maximum dorsiflexion during terminal stance unaffected/right side (°)	14.4 (4.0)	12.7 (4.0)	9.9 (3.4)	[2.8; 6.3]	[1.0; 4.4]	[-0.3; 3.9]
Maximum plantarflexion during pre-swing affected/left side (°)	13.1 (7.8)	15.5 (8.8)	19.5 (5.3)	[3.4; 9.3]	[0.9; 7.0]	[-2.1; 6.9]
Maximum plantarflexion during pre-swing unaffected/right side (°)	14.8 (8.0)	16.7 (6.7)	20.2 (4.9)	[2.5; 8.3]	[0.9; 6.1]	[-2.0; 5.8]
Range of motion during stance affected/left side (°)	23.8 (3.5)	22.1 (5.7)	19.7 (4.6)	[2.0; 6.1]	[0.1; 4.7]	[-0.9; 4.2]
Range of motion during stance unaffected/right side (°)	22.4 (4.7)	20.9 (4.3)	18.8 (4.6)	[1.4; 5.8]	[0.1; 4.2]	[-0.9; 3.9]
Range of motion during terminal stance/pre-swing affected/left side (°)	28.9 (7.7)	29.6 (8.4)	30.3 (5.6)	[-4.4; 1.7]	[-3.7; 2.4]	[-5.0; 3.7]
Range of motion during terminal stance/pre-swing unaffected/right side (°)	29.2 (7.3)	29.4 (7.3)	30.1 (5.3)	[-3.8; 2.0]	[-3.6; 2.0]	[-4.0; 3.8]

Parameter	Patients with knee OA N=29	Patients with hip OA N=30	Asymptomatic controls N=54	Knee OA - controls 95% CI	Hip OA - controls 95% CI	Hip OA - knee OA 95% CI
Knee kinematics						
Flexion at initial contact affected/left side (°)	3.4 (3.8)	5.0 (3.2)	5.2 (3.7)	[-3.6; 0.1]	[-1.9; 1.4]	[-3.5; 0.4]
Flexion at initial contact unaffected/right side (°)	4.8 (4.5)	6.2 (2.8)	5.7 (2.3)	[-2.4; 0.7]	[-0.6; 1.7]	[-3.4; 0.5]
Maximum flexion during weight acceptance affected/left side (°)	15.3 (5.9)	20.4 (7.0)	21.4 (5.1)	[-8.8; -3.4]	[-3.8; 1.8]	[-8.7; -1.6]
Maximum flexion during weight acceptance unaffected/right side (°)	19.0 (6.2)	22.3 (6.6)	22.0 (4.7)	[-5.6; -0.5]	[-2.3; 2.9]	[-6.7; 0.1]
Minimum knee flexion during pre-swing affected/left side (°)	8.4 (4.5)	15.3 (5.9)	9.3 (4.8)	[-3.3; 1.4]	[3.5; 8.5]	[-9.9; -4.0]
Minimum knee flexion during pre-swing unaffected/right side (°)	9.2 (5.5)	11.3 (5.1)	9.3 (4.0)	[-2.2; 2.1]	[-0.1; 4.1]	[-4.9; 0.7]
Maximum flexion during swing affected/left side (°)	58.0 (12.3)	64.7 (11.0)	68.1 (5.9)	[-14.5; -5.9]	[-7.3; 0.5]	[-13.1; -0.3]
Maximum flexion during swing unaffected/right side (°)	63.2 (9.0)	66.6 (8.8)	68.7 (5.3)	[-8.8; -2.3]	[-5.3; 1.1]	[-8.2; 1.3]
Range of motion during weight acceptance affected/left side (°)	7.0 (4.5)	5.1 (4.0)	12.1 (4.8)	[-7.5; -2.8]	[-9.1; -4.9]	[-0.5; 4.2]
Range of motion during weight acceptance unaffected/right side (°)	9.7 (5.5)	10.9 (5.8)	12.7 (4.0)	[-5.2; -0.8]	[-4.0; 0.5]	[-4.2; 1.8]
Range of motion during terminal stance/pre-swing affected/left side (°)	49.6 (10.7)	49.4 (8.5)	58.8 (5.3)	[-13.0; -5.5]	[-12.6; -6.3]	[-5.0; 5.5]
Range of motion during terminal stance/pre-swing unaffected/right side (°)	53.9 (7.0)	55.3 (6.1)	59.4 (4.6)	[-8.2; -2.8]	[-6.6; -1.7]	[-4.8; 2.1]

Parameter	Patients with knee OA N=29	Patients with hip OA N=30	Asymptomatic controls N=54	Knee OA - controls 95% CI	Hip OA - controls 95% CI	Hip OA - knee OA 95% CI
Hip kinematics						
Flexion at initial contact affected/left side (°)	29.0 (4.5)	22.7 (5.8)	29.9 (7.2)	[-3.8; 2.2]	[-10.3; -4.1]	[3.6; 9.2]
Flexion at initial contact unaffected/right side (°)	28.8 (5.3)	29.6 (9.0)	30.4 (5.4)	[-4.1; 0.9]	[-4.0; 2.4]	[-4.8; 3.1]
Maximum flexion during weight acceptance affected/left side (°)	29.6 (4.7)	23.8 (5.9)	31.1 (6.7)	[-4.4; 1.4]	[-10.3; -4.4]	[3.0; 8.7]
Maximum flexion during weight acceptance unaffected/right side (°)	29.7 (5.4)	30.8 (8.9)	31.4 (5.3)	[-4.2; 0.7]	[-3.8; 2.5]	[-5.1; 2.8]
Maximum extension during pre-swing affected/left side (°)	6.1 (3.0)	5.0 (3.3)	8.3 (3.5)	[0.6; 3.8]	[1.7; 4.8]	[-2.8; 0.6]
Maximum extension during pre-swing unaffected/right side (°)	5.4 (2.6)	5.9 (3.2)	8.4 (3.1)	[1.6; 4.3]	[1.1; 4.0]	[-1.1; 2.0]
Maximum flexion during swing affected/left side (°)	31.1 (4.7)	25.9 (6.1)	33.0 (8.1)	[-5.3; 1.5]	[-10.4; -3.6]	[2.2; 8.1]
Maximum flexion during swing unaffected/right side (°)	31.8 (4.9)	33.1 (9.8)	32.9 (4.8)	[-3.4; 1.2]	[-3.1; 3.4]	[-5.4; 2.9]
Range of motion during stance affected/left side (°)	35.7 (5.5)	28.8 (6.7)	39.5 (8.0)	[-7.1; -0.3]	[-14.1; -7.1]	[3.6; 10.2]
Range of motion during stance unaffected/right side (°)	35.1 (6.4)	36.7 (10.2)	39.8 (5.1)	[-7.3; -2.1]	[-6.5; 0.2]	[-6.1; 3.0]
Range of motion during pre-swing/swing affected/left side (°)	37.2 (5.4)	31.0 (6.1)	41.3 (9.7)	[-8.1; -0.1]	[-14.2; -6.4]	[3.1; 9.3]
Range of motion during pre-swing/swing unaffected/right side (°)	37.3 (5.8)	39.0 (10.4)	41.3 (5.0)	[-6.5; -1.6]	[-5.8; 1.0]	[-6.2; 2.8]
